# Potential Cause-and-Effect Relationship between Gut Microbiota and Childhood Neuroblastoma: A Mendelian Randomization Analysis

**DOI:** 10.1007/s12098-024-05065-6

**Published:** 2024-03-27

**Authors:** Jing Chu

**Affiliations:** Department of Pathology, Anhui Provincial Children’s Hospital, 39 Wangjiang East Road, Hefei, Anhui 230051 China

**Keywords:** Neuroblastoma, Gut microbiota, Mendelian randomization, GWAS, Single-nucleotide polymorphism

## Abstract

**Objectives:**

To analyze the potential causal-effect of gut microbiota (GM) on neuroblastoma (NB) risk using a Mendelian randomization (MR) study.

**Methods:**

A two-sample MR study was conducted using summary statistics of the GM from the largest available meta-analysis of genome-wide association studies conducted by the MiBioGen consortium. Pooled statistics for childhood NB were obtained from the IEU Consortium release data (1627 cases and 3254 controls). Inverse variance-weighted, weighted median, MR-Egger, and weighted mod were used to examine the causal relationship between GM and childhood NB. Single-nucleotide polymorphism (SNP) genes of positive GM were extracted using the PLINK program, and correlations between key SNP genes and tumor-regulated genes were analyzed. Functional enrichment analysis and transcription factor prediction were performed.

**Results:**

Inverse variance weighted (IVW) results indicated that *Erysipelotrichia* exerted a protective effect against childhood NB (odds ratio = 0.371, 95% Confidence interval: 0.173 - 0.795, *P* = 0.011) and that *Oscillospira* exerted a risk effect against childhood NB (odds ratio = 2.378, 95% Confidence interval: 1.121 - 5.043, *P* = 0.024), indicating the association of GM with childhood NB. Further screening analysis using the IVW test revealed a reliable causal relationship between *Erysipelotrichia* and NB. Two SNP genes (MUC4 and PELI2) of *Erysipelotrichia* were extracted and analyzed. Both key genes were significantly associated with tumor-regulated genes, enriched in several pathways associated with tumor progression, and correlated with several upstream transcription factors.

**Conclusions:**

It was observed that *Erysipelotrichia* is causally associated with NB using a two-sample MR study. Furthermore, the discovery of two SNP genes, MUC4 and PELI2, provides potential targets for the diagnosis and treatment of NB.

**Supplementary Information:**

The online version contains supplementary material available at 10.1007/s12098-024-05065-6.

## Introduction

Neuroblastoma (NB) is the most common extracranial solid tumor in children and accounts for 15% of childhood cancer-related deaths [1]. At the time of diagnosis, approximately 50% of patients have lymph node, liver, and bone marrow metastases with a poor prognosis [2]. Despite these multimodal treatments, the long-term survival rate of high-risk NB remains less than 40%, and the survival rate after relapse is less than 10% [3]. Managing high-risk patients remains challenging, and further clinical studies are needed to improve the treatment outcomes.

A growing body of evidence suggests a potential link between gut microbiota (GM) and cancer risk. The relationship between GM and NB in children has gradually become a focus of scientific research in recent years. Gut microorganisms can significantly influence the immune system by interacting with the host, especially T- cell differentiation and function. Thus, they indirectly regulate the anti-tumor immune response of the body. Meanwhile, bioactive molecules produced by certain intestinal bacteria are associated with tumor cell apoptosis, proliferation, and differentiation [4, 5]. In addition, the state of the GM may affect the absorption and metabolism of chemotherapeutic agents, thus indirectly influencing the efficacy and side effects of treatment [6]. These findings explain a possible direct relationship between gut microbiota and NB in children.

Mendelian randomization (MR) provides a novel approach for investigating potential causal associations between gut microbes and childhood NB. Using genetic differences as an instrumental variable for exposure, MR assists in assessing the direct relationship between specific exposures and disease outcomes. The transmission mode based on genetic information makes conclusions drawn through MR less susceptible to other potential confounders, thus enhancing the confidence of causal inferences [7, 8]. Here, the author has used pooled data from genome-wide association studies (GWAS) provided by the MiBioGen and IEU databases for MR analysis, aiming to clarify the relationship between gut microbes and pediatric NB.

## Material and Methods

1. Exposure data: GM genetic variation was obtained from the MiBioGen database, the largest genome-wide analysis of GM composition published to date. In this study, the microbial composition was analyzed using the V4, V3 - V4, and V1 - V2 variable regions of the 16S rRNA gene and classified using a direct taxonomic hierarchy. In this study, phylum, class, order, family, and genus were used as the classification levels, respectively, for a total of 9 phyla, 16 classes, 20 orders, 32 families, and 119 genera. An average abundance of >1% was identified.
2. Outcome data: Participants in the outcome-related GWAS studies selected for this study were predominantly of European ancestry. Summary outcome data were obtained from the IEU Consortium. In the study, pediatric NB cases were defined by their corresponding International Statistical Classification of Diseases (ICD) codes, and 1,627 pediatric NB cases and 3,254 controls were available.
3. The TCGA database (https://portal.gdc.cancer.gov/), the largest database of cancer gene information, holds data including gene expression data, copy number variations, Single-nucleotide polymorphism (SNPs), and other data. The author downloaded the raw mRNA expression data of pediatric NB data for subsequent analysis. A total of 162 specimens were collected for subsequent analysis.

The MR Base database (http://app.mrbase.org/) contains extensive summary statistics from hundreds of GWAS studies. Relevant causal relationships in the gut microbiota were extracted from the outcome IDs screened through the MR Base database in the GWAS summary data (https://gwas.mrcieu.ac.uk/). SNPs associated with each subclass at the genome-wide significance threshold (*P* <1.0 × 10^–5^) were selected as potential instrumental variables (IVs), and linkage disequilibrium (LDs) between SNPs were calculated. Among the SNPs with R2 <0.001 (clumping window size = 10,000 kb), only the SNPs with the lowest *P* value were retained. Inverse variance weighted (IVW, using a meta-analysis approach combining Wald estimates for each SNP), MR Egger (based on the assumption that instrumental strengths are independent of direct effects [InSIDE]), weighted median (the weighted median method allows the correct estimation of causality when up to 50% of IVs are invalid), and weighted mode (weighted model estimation has a greater ability to detect causal effects, smaller bias, and a lower type I error rate than MR-Egger regression) represent the four statistical methods that were used sequentially to assess the reliability of causality for obtaining an overall estimate of the effect of the GM on childhood NB. Finally, the screened causal relationships were validated and analyzed using heterogeneity (Cochran's IVW Q test) and genetic diversity tests.

Gene collections were downloaded from the molecular signatures database and the Gene set variation analysis (GSVA) algorithm was used to comprehensively score each gene collection to assess the potential biological function changes of different samples. Patients were divided into high- and low-expression groups based on the expression of their key genes, and Gene set enrichment analysis (GSEA) further analyzed the signaling pathway differences between the two groups.

The author explored the regulatory relationships of transcription factors and important genes using Cistrome DB, in which the genome file was set to hg38 and the transcription start site was set to 10 kb and visualized using Cytoscape.

Human neuroblastoma tumor cell lines SK-N-BE2 (MYCN‑amplified) and SH-SY5Y (non‑MYCN amplified) were obtained from Wuhan Pricella. Cells were kept in Dulbecco’s modified Eagle’s medium supplemented with 10% fetal bovine serum (Wisent, Ottawa, ON, Canada) and 1% penicillin in humid conditions at 37°C with a 5% CO_2_ atmosphere. The RNA from the cell lines, SK-N-BE2 and SH-SY5Y, was extracted using TRIzol reagent (Invitrogen), and the RevertAid First-Strand cDNA Synthesis Kit (Thermo Fisher Scientific, Inc.) was used to synthesise cDNA. Quantitative real-time PCR (qRT-PCR) analysis was performed using SYBR Green (Takara).

Reliable MR analyses were based on three presuppositional assumptions: (1) the correlation assumption (instrumental variables are strongly associated with exposure and not directly with the outcome); (2) the independence assumption (instrumental variables cannot be correlated with confounders); and (3) exclusivity assumption (instrumental variables can only affect the outcome through exposure, and genetic pleiotropy was judged to be present when the IV could affect the outcome by other means). All statistical analyses were performed using the R language (version 4.2.2). All statistical tests were two-sided, and *P* <0.05 was considered statistically significant.

## Results

A schematic representation of the study protocol has been shown in Supplementary Fig. S1.

**Fig. 1:**
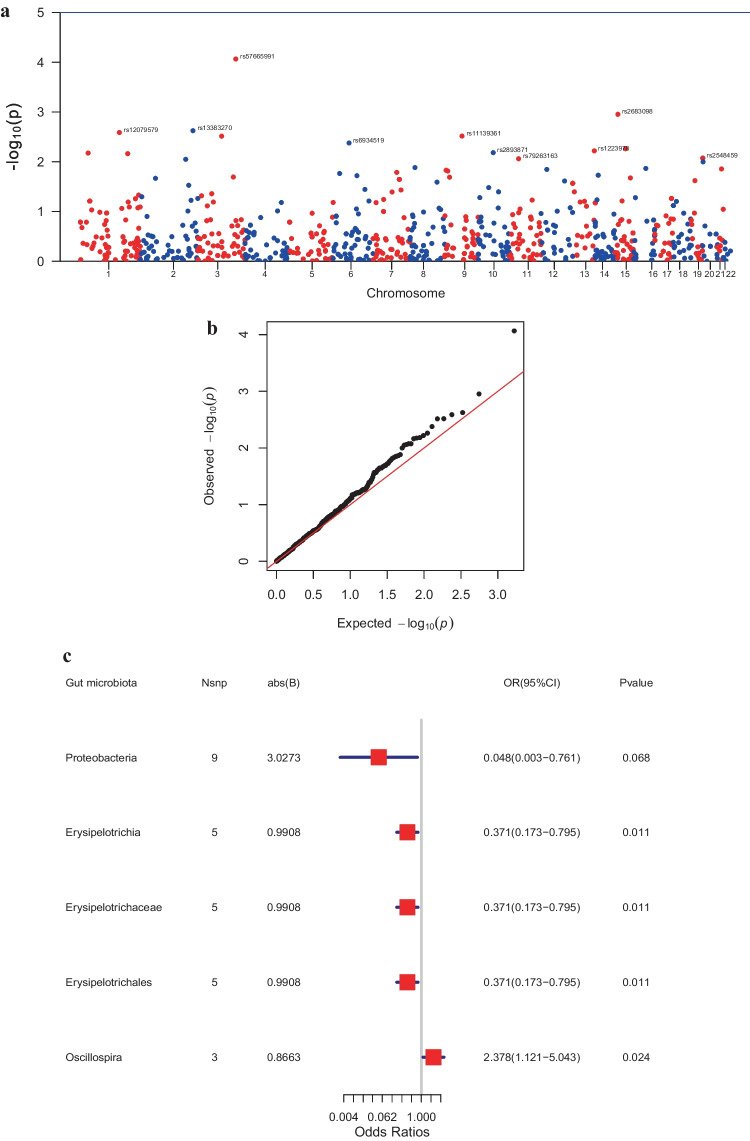

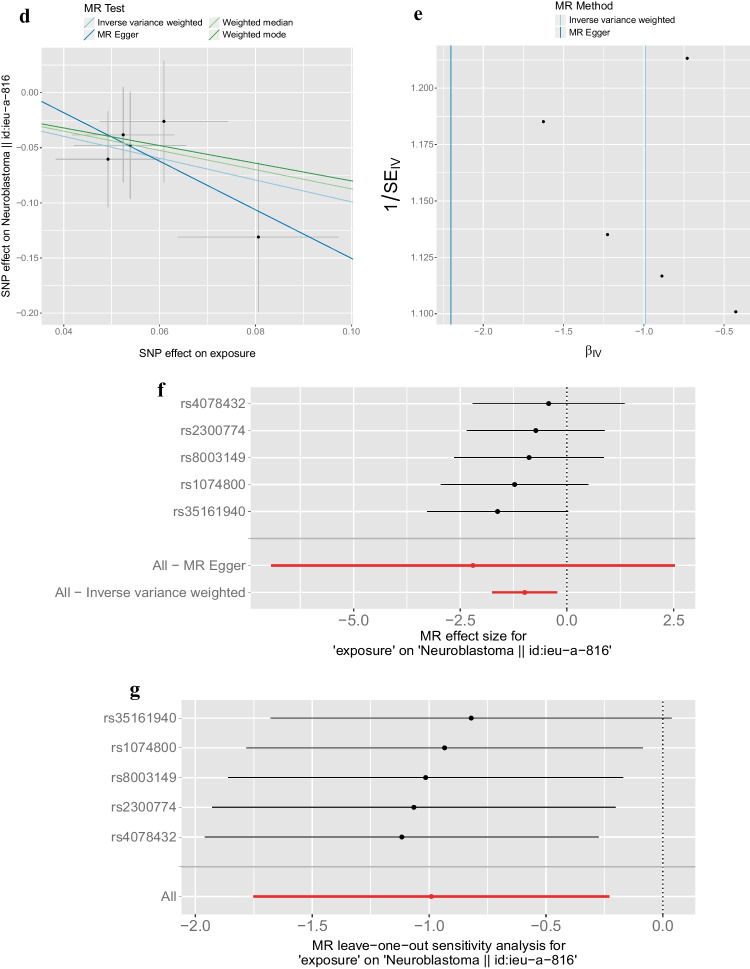
Mendelian randomization results. **(a)** Manhattan plot of GWAS analysis; **(b)** Quantile-Quantile plot of GWAS analysis; **(c)** Forest plot for summary causal effects of gut microbiota on NB risk; **(d)** Scatter plot of the MR analyses for the association of *Erysipelotrichia* and the risk of NB; **(e)** Funnel plot of heterogeneity analysis; **(f)** Forest plot of heterogeneity analysis; **(g)** The leave-one-out sensitivity analysis assessed the associations between genera and NB. *GWAS* Genome-wide association study, *NB* Neuroblastoma

The outcome ID of ieu-a-816 (Fig. 1a, b) was obtained from the pooled statistics of 4881 (controls, 3254; cases, 1627) samples associated with NB in children. Further Mendelian randomization analyses were performed to obtain five optimal causal relationships under different categorical levels in the gut microbiota (Fig. 1c, Table 1), *e.g.,* the presence of *Oscillospira intestinalis* (2.378; 1.121 - 5.043; *P* = 0.024) may be associated with a high risk of childhood NB, and *Erysipelotrichia* (0.371; 0.173 - 0.795; *P* = 0.011) was associated with a low risk of childhood NB. Further screening analysis using the IVW test revealed that *Erysipelotrichia* corresponded to a reliable causal association between exposure ID and ieu-a-816 (Fig. 1d).

**Table 1:** Mendelian randomization estimates of the associations between gut microbiota and risk of NB

**Microbiota**	**nsnp**	**b**	***p*-value**	**OR**	**OR_lci95**	**OR_uci95**
*Proteobacteria*	9	-3.02731	0.068211	0.048446	0.003083	0.761371
*Erysipelotrichia*	5	-0.99077	0.010775	0.371291	0.17337	0.795163
*Erysipelotrichaceae*	5	-0.99077	0.010775	0.371291	0.17337	0.795163
*Erysipelotrichales*	5	-0.99077	0.010775	0.371291	0.17337	0.795163
*Oscillospira*	3	0.866318	0.023895	2.378139	1.121431	5.043152

*NB* Neuroblastoma

The results of the heterogeneity analysis showed no heterogeneity in the causal relationships (Fig. 1e, f, Supplementary Table S1, *P* >0.05). Sensitivity analysis was performed using the leave-one-out cross-validation method, and the results showed that the effect on the overall error line after eliminating any of the SNPs was insignificant, indicating that the selected causal relationships were robust (Fig. 1g, Table 2). Finally, the PLINK program extracted the SNP-related genes in the TCGA expression profile in the following order: MUC4 and PELI2 (Table 3).

**Table 2:** MR leave-one-out sensitivity analysis for 'exposure' on NB

**SNP**	**chr**	**pos**	**effect_allele.outcome**	**b**	**se**	***p***
rs1074800	5	3004171	A	-1.226287714	0.881003827	0.163946327
rs2300774	3	195800811	G	-0.729916118	0.824245623	0.375856529
rs35161940	17	70334286	T	-1.625575773	0.84377268	0.054034919
rs4078432	14	48997206	C	-0.427570132	0.908389464	0.637861894
rs8003149	14	56160904	C	-0.8876358	0.895507031	0.321582898

*MR* Mendelian randomization, *NB* Neuroblastoma

**Table 3:** The PLINK program extracted the SNP-related genes in the TCGA

**CHR**	**SNP**	**BP**	***P*** **ANNOT**
3	rs2300774	195793712	8.95466339032433e-07 MUC4(0)| = missense
17	rs35161940	70327224	1.84509546544161e-06
14	rs8003149	56156504	4.08475070727283e-06 PELI2(0)
14	rs4078432	48997206	4.23103750003848e-06
5	rs1074800	3002546	6.14583285242795e-06

*SNP* Single-nucleotide polymorphism

**Fig. 2:**
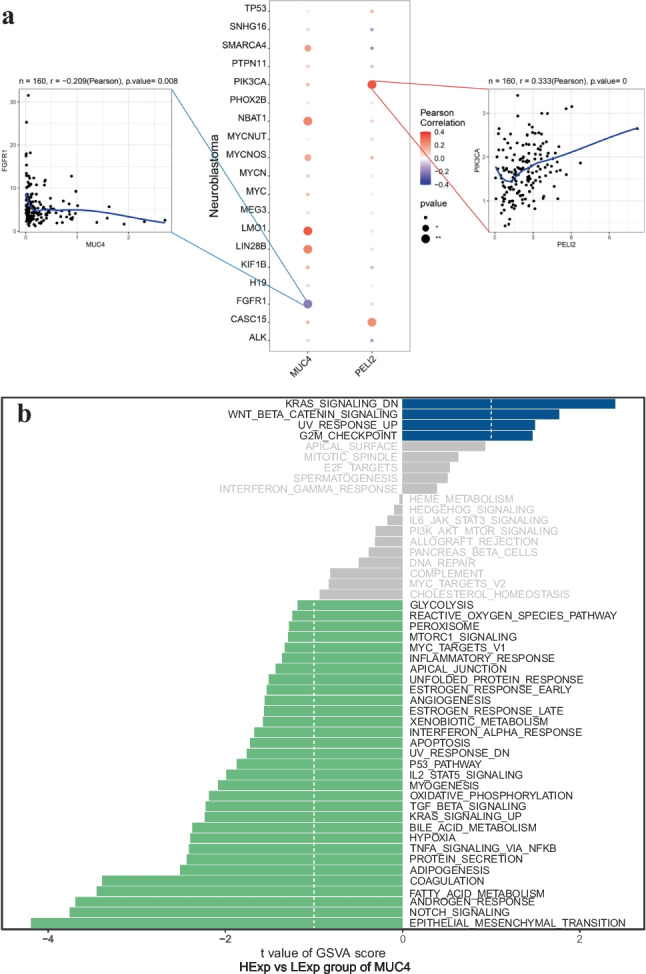

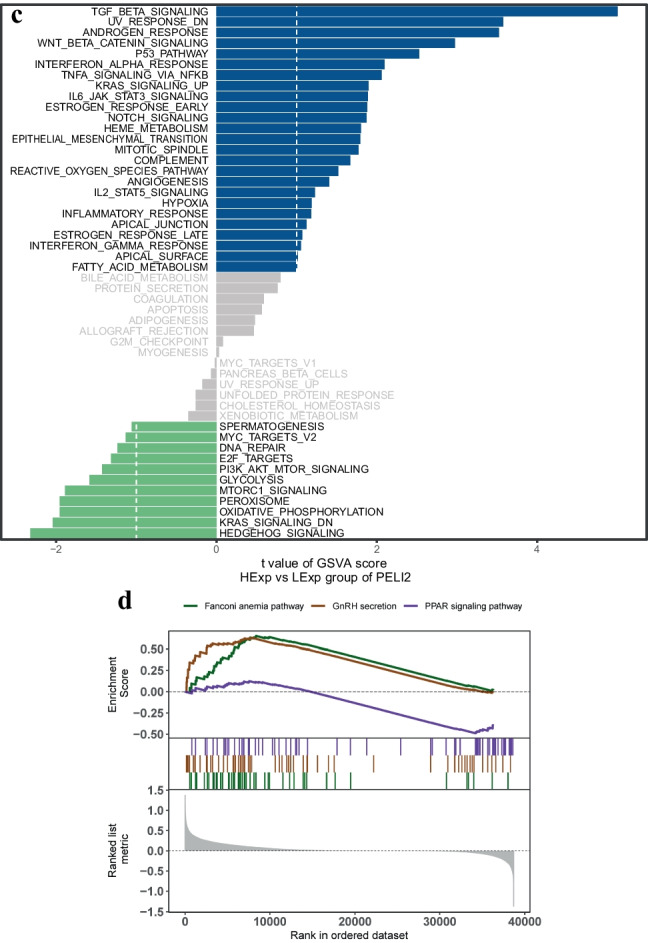

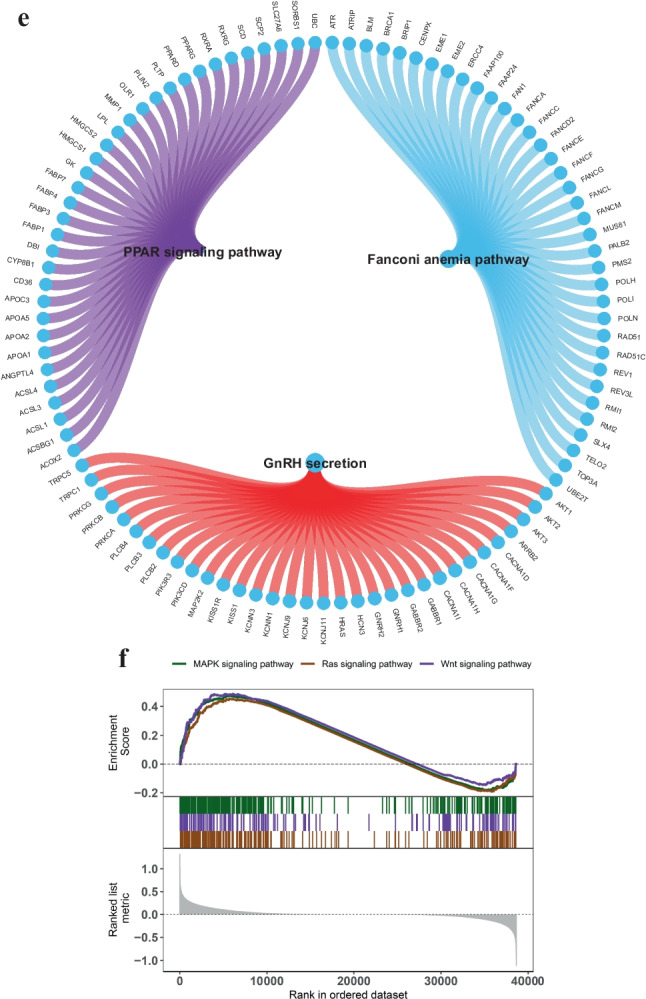

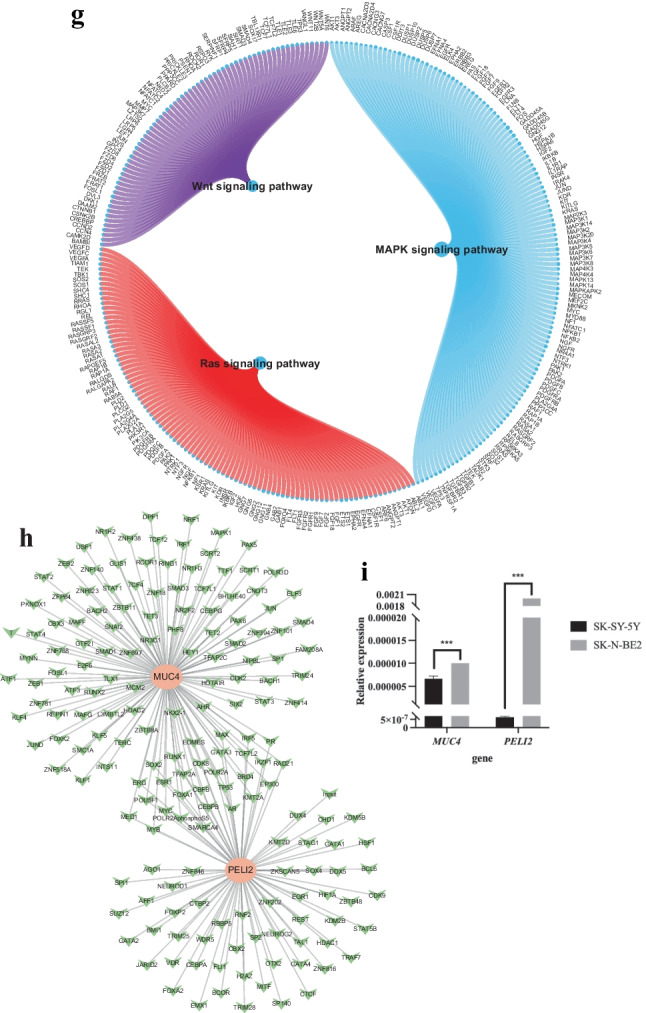
Exploring the regulatory network of SNP-gene effect relationships in NB. **(a)** The expression levels of SNP genes were significantly correlated with the expression levels of several disease-associated genes; **(b)** MUC4 of GSVA analysis; **(c)** PELI2 of GSVA analysis; **(d, e)** MUC4 of GSEA analysis; **(f, g)** PELI2 of GSEA analysis; **(h)** Transcriptional regulatory network of childhood NB-associated SNP genes; **(i)** Relative expression levels of MUC4 and PELI2 in SH-SY5Y and SK-N-BE2 cells. ***P <0.001 *vs.* SH-SY5Y cells. *GSEA* Gene set enrichment analysis, *GSVA* Gene set variation analysis, *NB* Neuroblastoma, *SNP* Single nucleotide polymorphism

The author obtained disease-causing genes associated with childhood NB from the GeneCards database (https://www.genecards.org/) and analyzed the expression levels of two SNP genes and the expression levels of the top 20 genes in the Relevance score and observed that the expression levels of SNP genes were significantly correlated with the expression levels of several disease-associated genes, among which PELI2 was significantly positively correlated with PIK3CA (cor = 0.333) and MUC4 was significantly negatively correlated with FGFR1 (cor =  −0.209) (Fig. 2a, Supplementary File S1).

The results of GSVA showed that the high expression of MUC4 was mainly enriched in the signaling pathways such as WNT_BETA_CATENIN_SIGNALING and KRAS_SIGNALING_DN (Fig. 2b), and the high expression of PELI2 was mainly enriched in the signaling pathways such as TGF_BETA_SIGNALING and P53_PATHWAY (Fig. 2c). In addition, GSEA analysis showed that MUC4 was enriched in Fanconi anemia, GnRH secretion, PPAR signaling, and other pathways (Fig. 2d, e); PELI2 was enriched in MAPK, Ras, Wnt, and other signaling pathways (Fig. 2f, g).

Two SNP genes were used for the gene set of this analysis to explore further the transcriptional regulatory networks involved in SNP genes. Relevant transcription factors were predicted using the Cistrome DB online database, of which 121 transcription factors were predicted for MUC4 and 84 transcription factors were predicted for PELI2. The transcriptional regulatory network of SNP genes associated with childhood NB was constructed by visualization using Cytoscape (Fig. 2h). Finally, the expression of MUC4 and PELI2 were assessed in the NB cell lines by qPCR. The primer sequences are summarized in Supplementary Table S2. SK‑N‑BE2 cells exhibit MYCN amplification and are known to be more aggressive than non-MYCN-amplified SH-SY5Y cells. As shown in Fig. 2i, the expression level of MUC4 and PELI2 in SK-N-BE2 cells (MYCN-amplified) was considerably higher than that in SH-SY5Y cells (non-MYCN amplified).

## Discussion

This is the first large-scale comprehensive MR study to investigate the causal relationship between gut microbiota and NB at the genetic prediction level. Accumulating evidence has suggested a potential link between the gut microbiota and cancers of other systems, including the breast, endometrial, lung, ovarian, and prostate [9]. Using an MR design, the author found that a higher genetically proxied abundance of the genus *Oscillospira* predicted a higher risk of NB. *Erysipelotrichia* was associated with a low risk of NB in children. Furthermore, IVW tests showed that the causal relationship between *Erysipelotrichia* and NB was more reliable. *Erysipelotrichia*, which produce short-chain fatty acids in the gut, including butyrate, have been associated with host lipid metabolism and diseases in humans [10]. MRx0029, a gut bacterium endemic in healthy humans, has been identified to induce differentiation phenotypes in undifferentiated neuroblastoma cells. This induction is facilitated through the production of C4–C6 fatty acids, particularly butyric, valeric, and hexanoic acids, leading to the upregulation of microtubule-associated protein 2 (MAP2) and synaptophysin (SYP). Microscopic examination revealed that SH-SY5Y cells treated with MRx0029 resembled a neuron-like phenotype, with multiple elongated processes on an oblong body [11]. In this study, *Erysipelotrichia* had a causal relationship with low-risk NB, which may be related to the neuronal differentiation effects of short-chain fatty acids.

*Oscillospira* showed a strong negative correlation with the Monocyte Chemoattractant Protein-1 (MCP-1) [12]. MCP-1 is a member of the C-C motif chemokine family and it functions as a potent agonist for monocytes, memory T cells, and basophils [13]. In NB, a surge in the abundance of *Oscillospira* results in a diminished expression of MCP-1. This reduction impairs the recruitment of immune cells, such as monocytes/macrophages and T cells, to the tumor site, thereby fostering an immunosuppressive tumor microenvironment and undermining the efficacy of tumor immunotherapies. The main finding of the present study implicates that stool examination may be a feasible strategy to identify populations at lower and higher risk of NB and to undertake more thorough examinations.

Next, the author extracted SNP-related genes from TCGA expression profile using the PLINK program, including MUC4 and PELI2. These two key genes were significantly related to NB tumor regulatory genes and were enriched in multiple tumor progression-related pathways and multiple upstream transcription factors. Mucin-4 (MUC4) is a secreted glycoprotein [14]. Lee et al. analyzed the whole exome sequencing data of 58 cases of MYCN non-amplified NB and found that mutations in mucin family genes, such as MUC4, MUC16, and MUC17, were more frequent in subjects with non-amplified MYCN [15]. In this study, MUC4 was significantly negatively correlated with FGFR1, which regulates cell-cell adhesion and extracellular matrix architecture and acts as an oncogene in NB [16]. These results suggest that MUC4 may act as a tumor suppressor in NB. PELI2 (also known as Pellino2) encodes an E3 ubiquitin ligase, which regulates the activation of nuclear factor kappa enhancer binding protein and MAPK cascades downstream of the TLR signaling pathway [17]. The GSVA findings reveal that high expression groups of MUC4 and PELI2 are intricately linked with the Wnt/β-catenin and KRAS signaling pathways. The aberrant activation of the Wnt/β-catenin pathway is critically involved in the pathogenesis of various cancers, including NB [18]. Investigations have illuminated that the constituents of the Wnt/β-catenin signaling pathway, including the activation of Wnt ligands such as WNT1, WNT2, and WNT5A, and the maladjustment of β-catenin, a pivotal downstream effector, contribute to NB progression [19]. KRAS mutations are implicated in approximately 25% of human cancers. Recent research has highlighted that RAS mutations, frequently leading to the activation of the RAS-MAPK pathway and the progression of NB, are commonly observed in cases of relapsed and treatment-resistant NB [20]. The results of qRT-PCR showed that MUC4 and PELI2 were upregulated in the SK-N-BE2 cells, compared to the non-MYCN-amplified SH-SY5Y cells. Finally, the author constructed a transcriptional regulatory network associated with childhood NB based on MUC4 and PELI2 expression.

## Conclusions

In summary, this is the first MR analysis using two samples to investigate the possible causal connection between the gut microbiota (especially *Erysipelotrichia*) and NB. Second, the author extracted gut microbiota SNP-associated genes from TCGA expression profile using the PLINK program and explored the potential effects and mechanisms of key genes in pediatric NB, as well as the regulatory networks of these genes.

However, there are certain limitations to the conclusions of this study. First, while the majority of patients in the GWAS summary data used in this study were European, only a small number of gut microbiota data were obtained from other races, which may have led to biased estimates and affected universality. Second, the GWAS dataset does not explicitly provide information on tumor status and patient survival or mortality data that significantly constrains the ability to precisely define tumor status and further analysis. Third, the SNPs selected as IVs may still be influenced by potential horizontal pleiotropy, because factors, such as genetic inheritance, lifestyle, and environmental conditions, can affect the gut microbiome, resulting in minor variations that may not be fully captured by IVs. These results necessitate further exploration of the molecular mechanism, providing a new approach to NB diagnosis and treatment.

## Supplementary Information

Below is the link to the electronic supplementary material.

Supplementary file1 (XLS 1 KB)

Supplementary file2 (DOCX 17 KB)

Supplementary file3 (DOCX 263 KB)
