# Supplementary material for: Potential Cause-and-Effect Relationship between Gut Microbiota and Childhood Neuroblastoma: A Mendelian Randomization Analysis
Source: Indian J Pediatr. 2024 Mar 27;92(7):717–24. doi: 10.1007/s12098-024-05065-6 (PMC12182494; doi:10.1007/s12098-024-05065-6)
Supplement: Supplementary file 2 — Supplementary file2 (DOCX 17 KB) [file 12098_2024_5065_MOESM2_ESM.docx]

**Supplementary Table S1.** The heterogeneity results from the Cochran’s Q test

| **id.exposure** | **id.outcome** | **Outcome** | **Exposure** | **Method** | **Q** | **Q_df** | **Q_*p* val** |
| --- | --- | --- | --- | --- | --- | --- | --- |
| ebi-a-GCST90016916 | ieu-a-816 | Neuroblastoma \|\| id:ieu-a-816 | exposure | MR Egger | 0.876742673 | 3 | 0.831036398 |
| ebi-a-GCST90016916 | ieu-a-816 | Neuroblastoma \|\| id:ieu-a-816 | exposure | Inverse variance weighted | 1.135302046 | 4 | 0.888631458 |

**Supplementary Table S2.** Primer sequences used for reverse transcription-quantitative PCR

| **Gene** | **Primer sequences (5'→3')** |
| --- | --- |
| MUC4 | F: AGGACGCACCCACCATAAGT  R: GTTTTGGAAAGTGACGTGCC |
| PELI2 | F: CGTGTGCGACAGGAATGAAC  R: CATCACCAGGACGCCATTAG |
| GAPDH | F: GAAGGTGAAGGTCGGAGTCAA  R: CTGGAAGATGGTGATGGGATTT |

*F* Forward; *R* Reverse
