# Supplementary figures and images for: Potential Cause-and-Effect Relationship between Gut Microbiota and Childhood Neuroblastoma: A Mendelian Randomization Analysis
Source: Indian J Pediatr. 2024 Mar 27;92(7):717–24. doi: 10.1007/s12098-024-05065-6 (PMC12182494; doi:10.1007/s12098-024-05065-6)

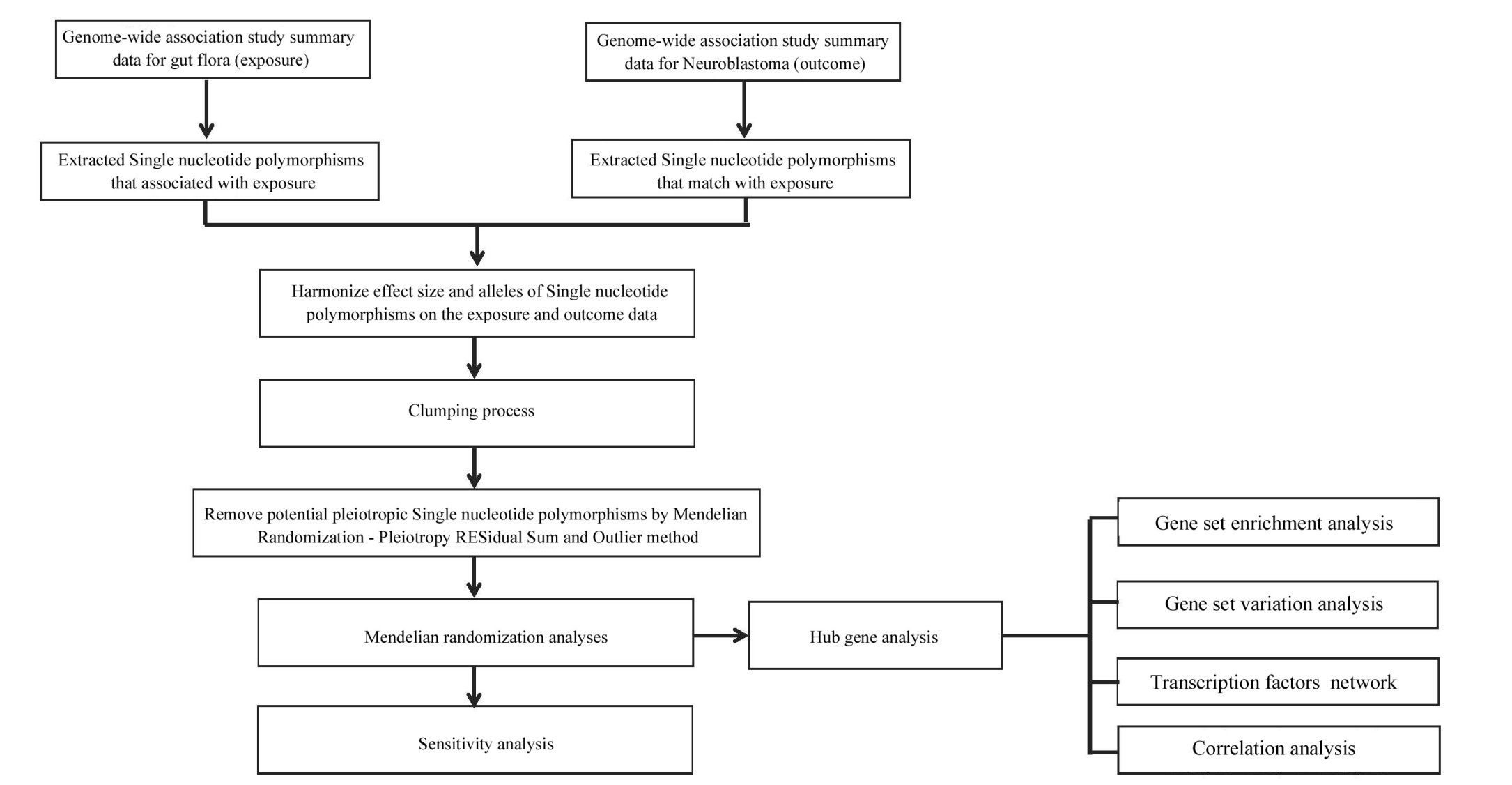


**Supplementary Fig. S1** The flow chart describing the research idea and content of this study.

Supplement: Supplementary file 3 — Supplementary file3 (DOCX 263 KB) [file 12098_2024_5065_MOESM3_ESM.docx]
